# Role of *Porphyromonas gingivalis* outer membrane vesicles in oral mucosal transmission of HIV

**DOI:** 10.1038/s41598-018-27284-6

**Published:** 2018-06-11

**Authors:** Xin-Hong Dong, Meng-Hsuan Ho, Bindong Liu, James Hildreth, Chandravanu Dash, J. Shawn Goodwin, Muthukumar Balasubramaniam, Chin-Ho Chen, Hua Xie

**Affiliations:** 1 Department of Microbiology and Immunology, Meharry Medical College, Nashville, TN 37027, USA; 2 School of Dentistry, Meharry Medical College, Nashville, TN 37027 USA; 3 Department of Biochemistry and Cancer Biology, Meharry Medical College, Nashville, TN 37027 USA; 4 Department of Surgery, Duke University Medical Center, Durham, NC 27710 USA

## Abstract

The association between mucosal microbiota and HIV-1 infection has garnered great attention in the field of HIV-1 research. Previously, we reported a receptor-independent HIV-1 entry into epithelial cells mediated by a Gram-negative invasive bacterium, *Porphyromonas gingivalis*. Here, we present evidence showing that *P. gingivalis* outer membrane vesicles (OMVs) promote mucosal transmission of HIV-1. We demonstrated, using the Dynabeads technology, a specific interaction between HIV-1 and *P. gingivalis* OMVs which led to an OMV-dependent viral entry into oral epithelial cells. HIV-1 was detected in human oral keratinocytes (HOKs) after a 20 minute exposure to the HIV-vesicle complexes. After entry, most of the complexes appeared to dissociate, HIV-1 was reverse-transcribed, and viral DNA was integrated into the genome of HOKs. Meanwhile, some of the complexes exited the original host and re-entered neighboring HOKs and permissive cells of HIV-1. Moreover, *P. gingivalis* vesicles enhanced HIV-1 infection of MT4 cells at low infecting doses that are not able to establish an efficient infection alone. These findings suggest that invasive bacteria and their OMVs with ability to interact with HIV-1 may serve as a vehicle to translocate HIV through the mucosa, establish mucosal transmission of HIV-1, and enhance HIV-1 infectivity.

## Introduction

The human immunodeficiency virus (HIV)/AIDS pandemic continues to be a major global health problem, as millions of people worldwide are currently living with HIV/AIDS^1^. The majority of new HIV infections originate at the genital and rectal mucosa. The mechanism of HIV transmission through the mucosal epithelium remains unclear. Generally, epithelial surfaces present the first line of defense against pathogens such as bacteria and viruses. Nevertheless, HIV-1 can penetrate a multi-layer epithelium in the absence of any apparent breach or lesion^2,3^. Several mechanisms have been proposed to explain how HIV-1 gains access to permissive cells across mucosal surfaces^4^. First, HIV-1 may cross the epithelial layer by moving through intercellular compartments. Next, mucosal dendritic cells may facilitate HIV entry through the epithelium by capturing cell-free- or cell-associated HIV-1 using their extending dendrites within the epithelial layers. Finally, epithelial cells may take-up HIV-1 particles and transfer them to target cells such as dendritic cells, T-cells, and macrophages by a process known as transcytosis^5^.

Recently, the role of the microbiome in HIV transmission of mucosa has drawn great attention from HIV researchers. For example, Mirmonsef and Spear showed that lower levels of *Lactobacillus* in the genital tract are associated with an increased risk of acquiring HIV-1 and female-to-male transmission^6^. Another example is that *Porphyromonas gingivalis*, an invasive bacterium usually found in the oral cavity may play a role in HIV-1 transmission. A series of studies by Herzberg *et al*. revealed an up-regulated expression of CCR5 in oral epithelial cells induced by *P. gingivalis* LPS and gingipains. Consequently, *P. gingivalis* could selectively promote R5-tropic HIV-1 infection of oral keratinocytes and viral dissemination^7–9^. Previously, we reported a specific interaction between HIV-1 and *P. gingivalis* that is mediated by the binding domain (HGP44) of bacterial gingipains and the viral envelope glycoprotein, gp120^10^. We further demonstrated that invasive *P. gingivalis* strains promoted both R5-tropic and X4-tropic HIV-1 entry into epithelial cells by trapping the viruses on their surfaces and carrying the virions into the cells^11^.

Most Gram-negative bacteria secrete outer membrane vesicles (OMVs) as either surface-bound or cell-free spherical structures. OMVs have recently gained recognition for their role in bacterial virulence and are known to play similar roles as their parent cells in mediating adherence, biofilm formation, invasion, host cell damage, and modulation of host immune responses^12^. We previously reported that adhesive molecules such as gingipains and FimA were enriched in *P. gingivalis* OMVs and that the OMVs were much more invasive than their parent cells^13,14^. In the study presented here, we built upon our previous work on the receptor-independent HIV-1 entry mediated by P. gingivalis cells11. We further examined the ability of P. gingivalis vesicles to promote HIV-1 entry into and spreading among human oral keratinocytes (HOKs) as well as HIV-1 permissive cells. These findings provide insights into the potential mechanisms of mucosal HIV-1 transmission.

## Results

### The interaction of HIV-1 and *P. gingivalis* vesicles

**Figure 1:**
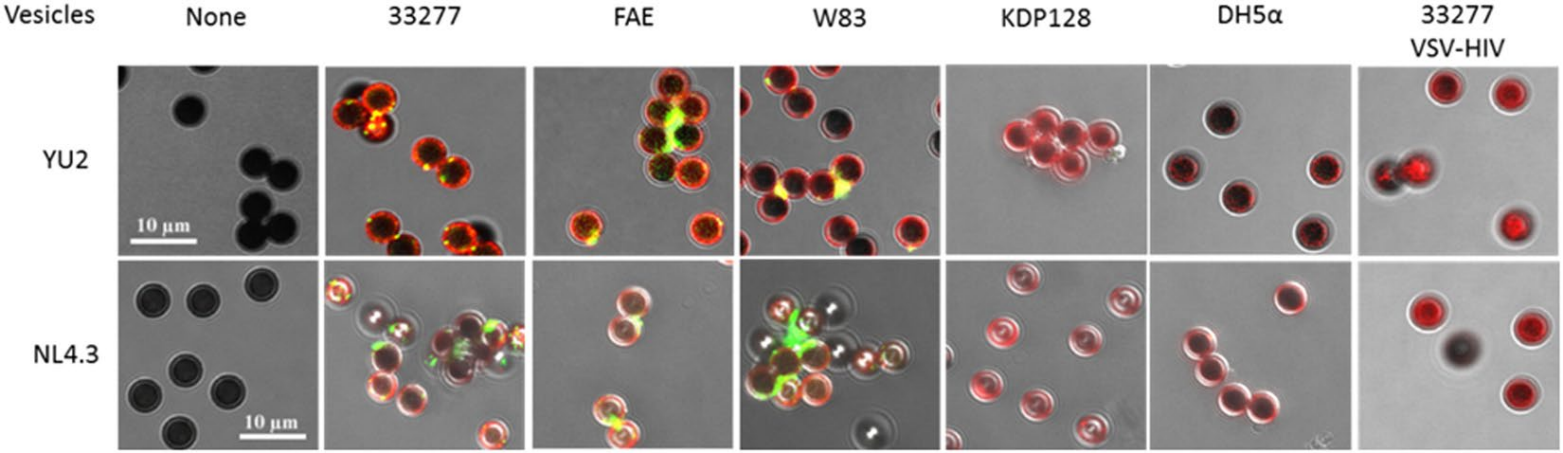
Specific binding of HIV-1 to *P*. *gingivalis* 33277 vesicles. Vesicles derived from *P*. *gingivalis* 33277 (wild type strain expressing FimA I protein), FAE (the *fimA* mutant), W83 (a type strain lacking FimA), KDP128 (a gingipain triple mutant) and *E. coli* DH5α were coupled with Dynabeads. The beads were then incubated with HIV_NL4.3_ or HIV_YU2._ Vesicles derived from 33277 were also exposed to VSV pseudotyped HIV (VSV-HIV). Images were acquired using a confocal microscope. Bacterial vesicles (red) were stained with polyclonal antibodies specific for *P. gingivalis* or *E.coli*, and Alex Fluor 546-conjugated secondary antibodies. HIV-1 virions (NL4.3 and YU2, green) were stained with anti-p24 monoclonal antibody and Alexa Fluor 488-conjugated anti-mouse IgG.

We previously reported that HIV-1 binds specifically to the surface of *P. gingivalis* through the interaction between the binding domains (HGP17 and HGP44) of gingipains (an important class of *P. gingivalis* extracellular outer membrane-associated proteases) and HIV-1 gp120^10^. Therefore, we speculated that *P. gingivalis* may serve as a vehicle for carrying HIV-1 into cells that are non-permissive to HIV, such as oral epithelial cells. Recently, we demonstrated that HGP44 was enriched in *P. gingivalis* OMVs that were much more invasive than their parent cells^13,14^. We tested if *P. gingivalis* OMVs also specifically interact with HIV-1. Binding experiments were performed using magnetic beads coupled with *P. gingivalis* vesicles. Either X4 or R5 tropic HIV-1 (NL4.3 or YU2) were incubated with the beads and visualized with immunostaining and confocal microscopy. Binding of HIV-1 to beads was found on those coupled with vesicles derived from *P. gingivalis* strains 33277 (the *fimA* I strain), FAE (the *fimA* mutant), and W83 (the *fimA* IV strain) (Fig. 1), all of which express HGP44. We also observed that the HIV-bound beads tended to cluster together when HIV bound to the beads, possibly due to HIV bridging. Conversely, we did not detect the binding of NL4.3 and YU2 virions on beads coupled with vesicles from *P. gingivalis* KDP128, a triple mutant that does not express gingipains^15^, or on those coupled with *E. coli* DH5α vesicles. Moreover, VSV-G pseudotyped HIV lacking HIV-1 envelope proteins did not bind to beads coupled with 33277 vesicles. These results demonstrate a specific interaction between HIV-1 and *P. gingivalis* vesicles.

### *P. gingivalis* vesicle-dependent HIV-1 entry into non-permissive cells

**Figure 2:**
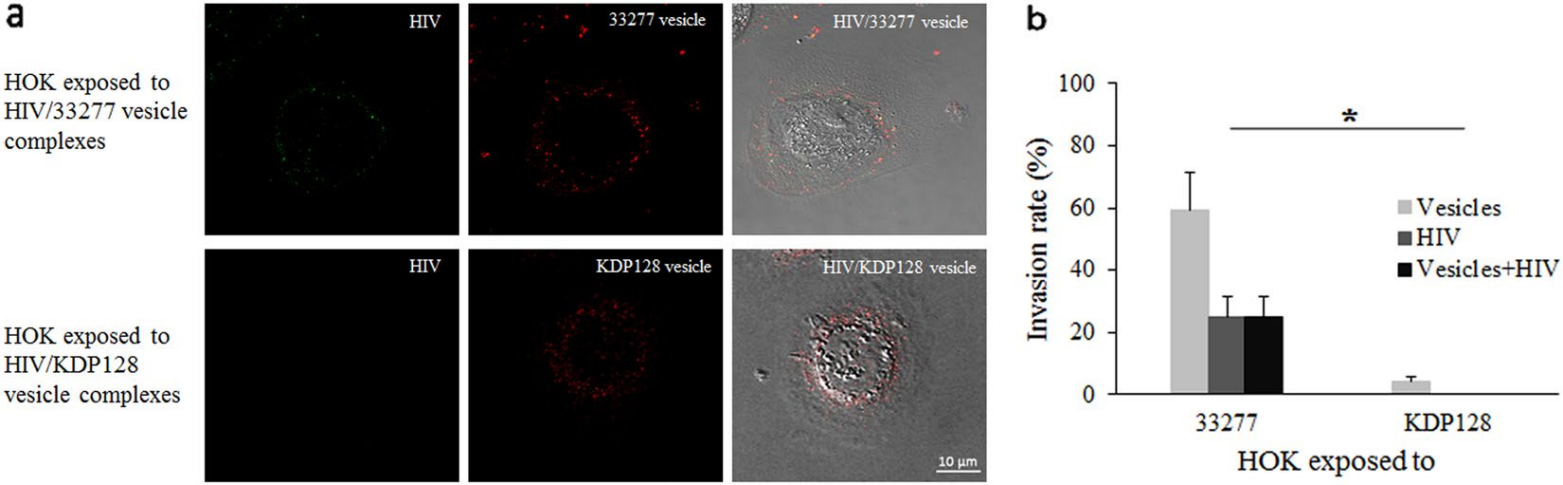
Effects of *P. gingivalis* vesicles on HIV-1 internalization of HOKs. HOKs were exposed to HIV-vesicle complexes for 30 min. (**a**) The pper panel was images of HOK exposed to HIV/*P. gingivalis* 33277 vesicle complexes, and the lower panel was HOK treated with HIV/the gingipain mutant KDP128 vesicle complexes. HIV_NL4.3_ (green) and *P. gingivalis* vesicles (red) were visualized in HOKs using confocal microscopy. HOKs were shown by differential interference contrast images (overlay) to show cell boundary and morphology. Scale bar represents 10 μm. (**b**) The numbers of HOKs with intercellular *P. gingivalis* vesicles were determined by counting ten random areas. Each bar represents the percentage of HOKs with intercellular vesicles or HIV-1. An asterisk indicates the statistical significance of invasive rates between 33277 vesicles and KDB128 vesicles (*P* < 0.05; *t* test).

**Figure 3:**
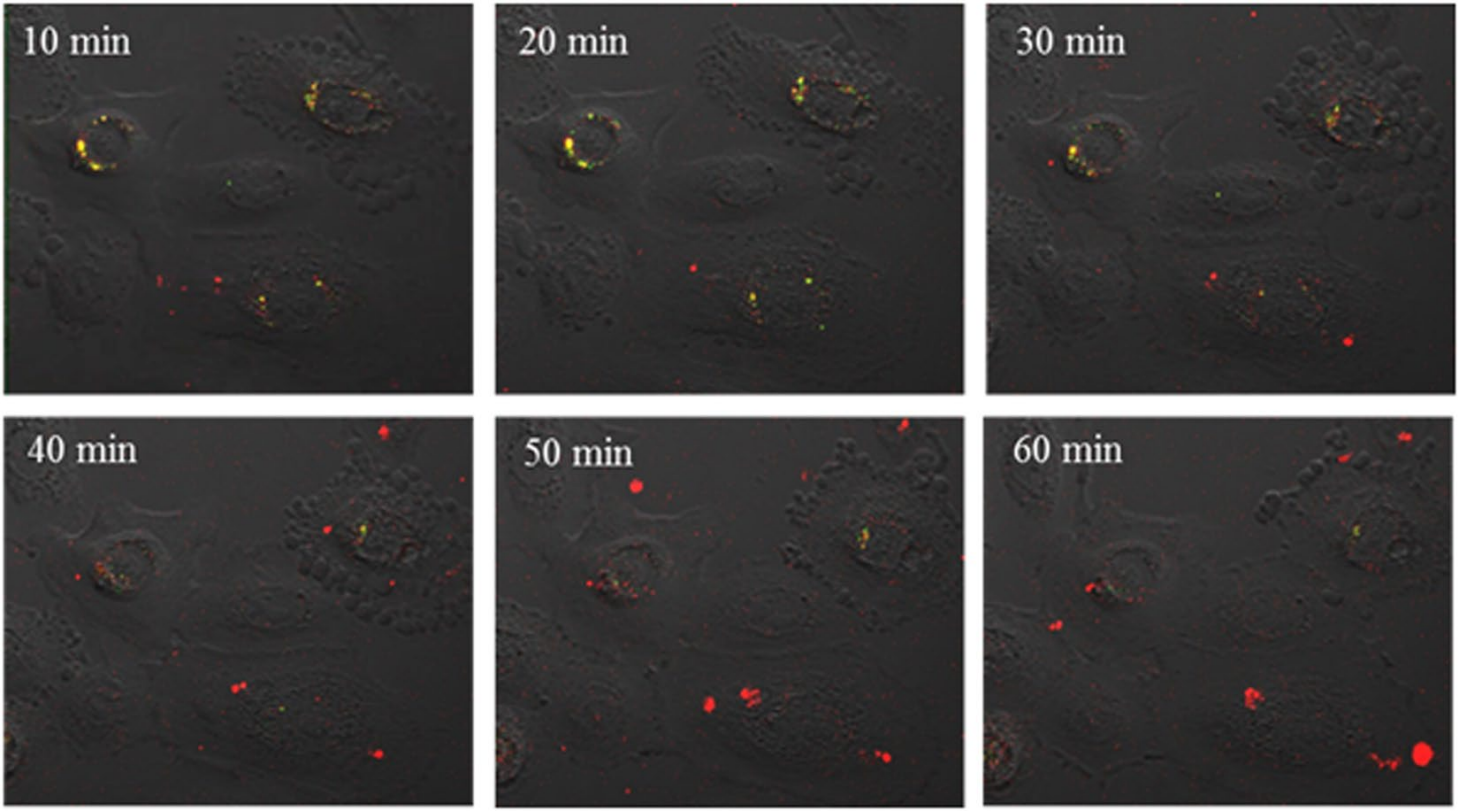
Time course for internalized HIV-*P. gingivalis* vesicle complexes in HOKs. HOKs were incubated with the Alexa Fluor 594 labeled vesicles (red) and GFP-tagged HIV (green) for 60 min at 37 °C. Images were obtained by confocal microscopy at 10, 20, 30, 40, 50, and 60 min.

To test if *P. gingivalis* vesicles can carry HIV-1 into non-permissive cells such as HOKs, we co-incubated the host cells with either HIV_NL4.3_ alone, HIV/*P. gingivalis* 33277 vesicle complexes, or HIV/KDP128 vesicle complexes for 30 minutes. Cell-free complexes were removed by washing the cells with PBS, and the cells were then fixed, permeabilized, immunostained, and analyzed using confocal microscopy. As shown in Fig. 2a, intracellular vesicles were mainly observed in the perinuclear area, which is in agreement with the previous report^16^. HIV_NL4.3_ (green) was internalized only in those cells that also carried intracellular *P. gingivalis* 33277 vesicles (red). Some HIV-1 particles still co-localized with *P. gingivalis* vesicles (yellow) after a 30 min exposure, while others were independent from 33277 vesicles, suggesting that at least some HIV-1 had dissociated from the bacterial vesicles after entering the host cells. In contrast, internalization of vesicle-free HIV-1 was not observed in HOKs exposed to HIV-1 alone (data not shown). We also did not find vesicle-free and vesicle-associated HIV-1 particles in HOKs exposed to the vesicles derived from *P. gingivalis* KDP128 (a gingipain negative mutant)/HIV-1 complexes. The vesicles of the gingipain mutant exhibited a decreased ability to invade HOKs, which is in agreement with the previous report on the role of gingipains in *P. gingivalis* invasion^17^. As expected, the vesicles of KDP128 could not promote HIV internalization. To qualify the invasion rates, we counted HOKs with internalized vesicles or HIV in six random areas (1.34 × 1.34 mm) (Fig. 2b). We found that 59.2% of the HOKs contained the internalized vesicles and 25% of HOKs with HIV-1 after exposed to 33277 vesicle/HIV complexes. The invasion of KDP128 vesicles was much lower (4% of HOKs, *P* < 0.001) than that of 33277 vesicles, and few HIV-1 particle was found in the HOKs exposed to KDP128 vesicle/HIV complexes, indicating that the observed HIV-1 entry into HOKs depends on HIV and vesicle interaction as well as invasive activity of the vesicles. To further confirm HIV-1 entry of HOKs, we performed live-cell imaging using fluorescently labeled HIV-1 and *P. gingivalis* 33277 vesicles. We observed a rapid entry of HIV-1/vesicle complexes into the HOKs. Within 10 min of incubation with HOKs, the complexes entered the cells and reached the perinuclear region (Fig. 3, Video S1). The majority of these complexes dissociated 40 min after entry, although some HIV-vesicle complexes remained in the cells 1 h post-entry. Previously, we demonstrated that Dynasore, an inhibitor of clathrin-dependent endocytosis, blocked *P. gingivalis* vesicle entry into HOKs^18^. As expected, we did not find HIV-vesicle complexes in the cytoplasm of HOKs in the presence of Dynasore (35 µM) (Video S2). These findings reveal an endocytosis-dependent HIV-1 entry of non-permissive cells, which is mediated by *P. gingivalis* vesicles.

### Outcomes of co-entry of HIV and OMVs into HOKs

**Figure 4:**
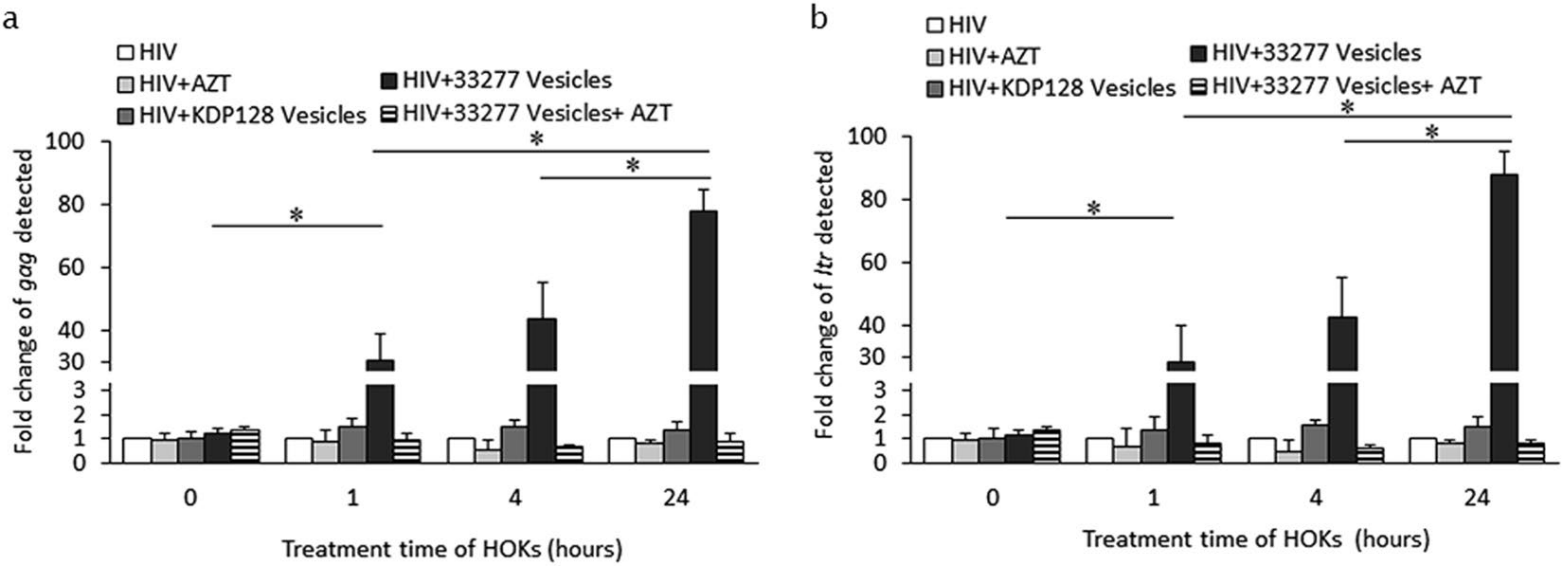
Detection of HIV reverse-transcription in HOKs. DNA levels of HIV_NL4.3_ *gag* (**a**) and *ltr* (**b**) in HOKs were measured using qPCR during a 24-hr period after the initial infection. Each bar represents the relative fold increase in HIV_NL4.3_ DNA level detected in HOKs exposed to HIV-vesicle complexes compared to that in unexposed cells (designated as 1 unit). Error bars represent SD (n = 3). Asterisks indicate statistically significant differences between HIV_NL4.3_ DNA levels in exposed and unexposed host cells (*P* < 0.05; *t* test).

As shown in Fig. 3, the internalized HIV-1 could either remain associated with *P. gingivalis* vesicles, or free itself from the vesicles. We speculated that HIV-vesicle complexes spread from the initial host epithelial cells to neighboring cells, while vesicle-free viruses may be uncoated and have their RNA reverse-transcribed. We first determined if we could detect viral DNA in HOKs exposed to either HIV_NL4.3_ alone or HIV-*P. gingivalis* 33277 vesicle complexes for 0, 1, 4, or 24 h, respectively. Reverse-transcribed viral DNA was identified using qPCR with HIV *gag* and *ltr* specific primers. There was little viral DNA detected in HOKs exposed to HIV-1 alone even after a 24 h exposure. On the other hand, reverse-transcribed viral DNAs were detected in the HOKs exposed to the HIV/vesicle complexes after only 1 h of exposure, and a significant increase in viral DNA was found in the HOKs exposed to the complexes for 4 and 24 h (Fig. 4a,b). Approximately 30 times more viral DNA was found in HOKs treated with HIV/vesicles complexes for 1 h (30 ± 8.18, *P* = with *gag* primers; 27 ± 11.1, *P* = 0.041, with *ltr* primers) when compared to that detected in HOKs exposed to HIV alone. At least 40 times (43 ± 11.6, *P* = 0.025 with *gag* primers; 42 ± 12.3, *P* = 0.002 with *ltr* primers) and 75 times (77 ± 6.63, *P* = 0.0018 with *gag* primers; 87 ± 7.63, *P* = 0.002 with *ltr* primers) more viral DNA were detected in HOKs after 4 or 24 h exposure. Treatment of HOKs with zidovudine (AZT, 15 µM), a reverse-transcriptase inhibitor, efficiently blocked HIV_NL4.3_ reverse-transcription in HOKs, and the viral DNA was not detected in the HOKs exposed to the complexes in the presence of AZT.

**Figure 5:**
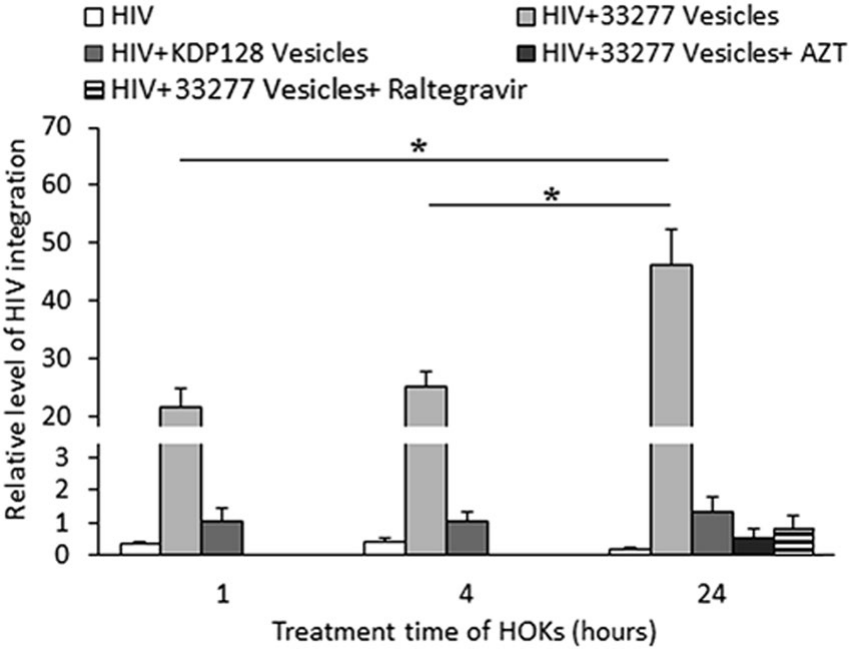
HIV-1 proviral DNA integration into the genomic DNA of HOKs. HOKs were incubated with either HIV_NL4.3_ alone or HIV/vesicle complexes for 1, 4, or 24 h, DNA were extracted, and levels of HIV provirus was determined using the Alu- LTR-based RT-PCR assay. As controls, HOKs also treated with either AZT or raltegravir. Data are represented as mean values, with error bars indicating SD of triplicate measurements. Asterisks indicate statistically significant differences between proviral DNA levels in HOKs exposed to HIV/vesicle complexes for 1, 4 and 24 h (*P* < 0.05; *t* test).

We further tested if the reverse-transcribed viral DNA was integrated into the genome of HOKs. Alu-LTR real-time nested PCR assays were perform using genomic DNAs isolated from HOKs treated with either HIV_NL4.3_ or HIV/vesicle complexes, in the presence or absence of HIV integrase inhibitor raltegravir (MK-0518, 10 µM). Integrated provirus was detected in the HOKs as early as 1 h after the cells were exposed to the HIV/vesicle complexes, its level increased by two-fold after 24 h (Fig. 5). Moreover, raltegravir treatment significantly blocked HIV integration, and provirus was also not found in the HOKs treated with AZT, apparently due to its inhibitory activity of reverse-transcriptase.

**Figure 6:**
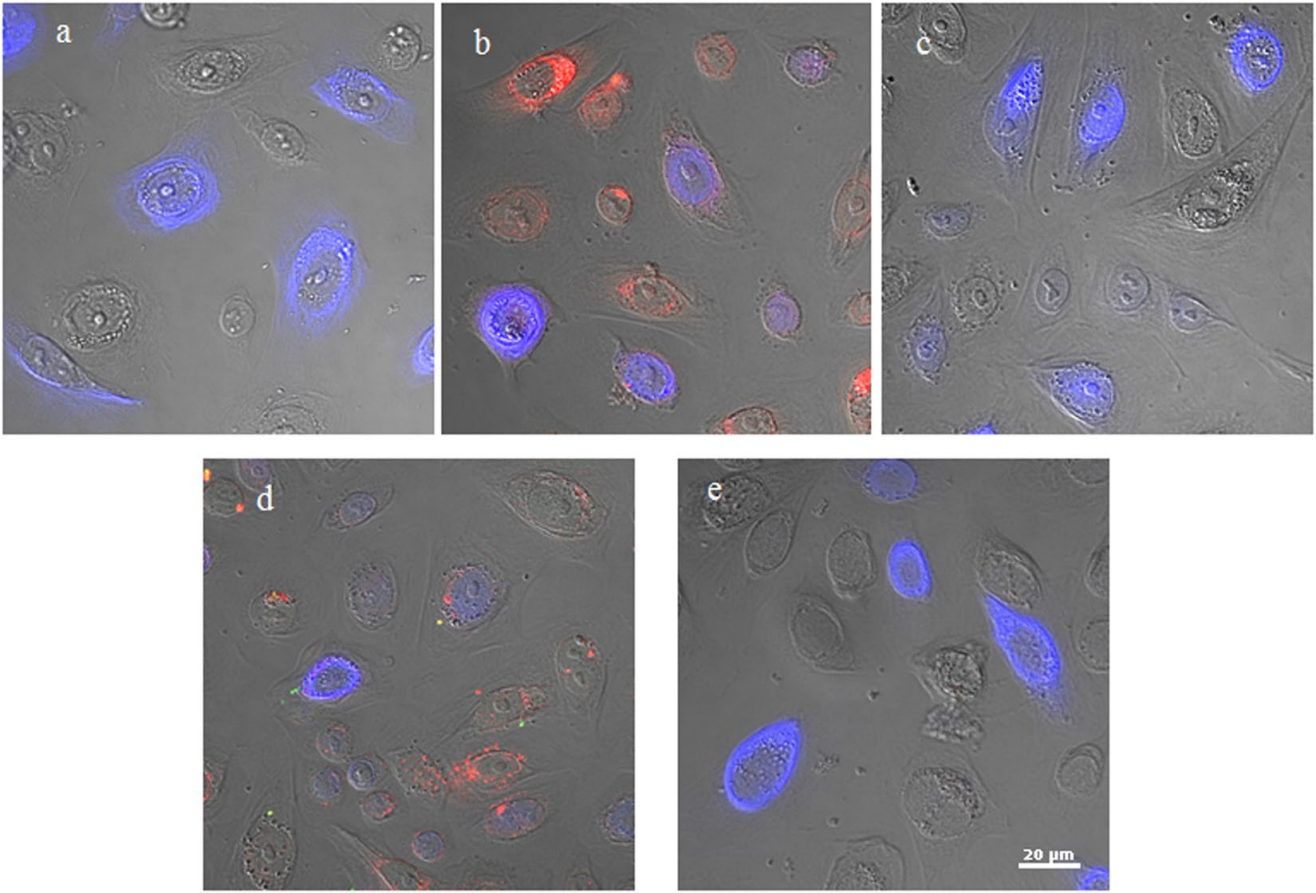
Effect of *P. gingivalis* vesicles on HIV_NL4.3_ spreading among HOKs. HOKs (unstained) were initially exposed to HIV_NL4.3_ alone, *P. gingivalis* 33277 vesicles alone, or HIV- vesicle complexes. These pretreated cells were then mixed and cultured with secondary HOKs that had not been exposed to the virus or vesicles (blue) for 24 h. Images were obtained by immunofluorescence confocal microscopy. (**a**) HOKs without exposure to neither HIV nor vesicles. (**b**) HOKs that were exposed to *P. gingivalis* vesicles (red) alone. (**c**) HOKs that were exposed to HIV_NL4.3_ alone. (**d**) HOKs that were initially exposed to HIV/vesicle complexes in the absence of Dynasore. (**e**) HOKs were pre-treated with Dynasore, and exposed to HIV-vesicle complexes. Scale bar, 20 μm.

To examine if internalized HIV-vesicle complexes are able to exit their original host cells and enter neighboring HOKs, we first treated HOKs with *P. gingivalis* 33277 vesicles alone, HIV_NL4.3_ alone, or HIV-vesicle complexes for 1 h. Cell-free HIV or HIV-vesicle complexes were removed, and the initially treated HOKs (5 × 10^4^ cells) were mixed with a new set of uninfected HOKs (5 × 10^4^) that were labeled with CellTracker. The mixed cells were analyzed by confocal microscopy. As shown in Fig. 6a–e, after *P. gingivalis* vesicles entered the initially treated HOKs, some of the vesicles exited and re-entered to neighboring cells. Apparently, HIV-vesicle complexes were also observed in the CellTracker labeled HOKs after being mixed with HOKs initially treated with HIV-vesicle complexes, suggesting a vesicle-dependent spreading of HIV among HOKs. To exclude the possibility that the HIV and vesicles detected in the CellTracker labeled HOKs is due to extracellular HIV-1 and vesicles that were not removed after washings, we pre-treated the initially exposed HOKs with dynasore (30 µM). Previously, we showed that Dynasore efficiently prevented *P. gingivalis* cells and their OMVs from entering into HOKs^18^. As expected, HIV-vesicle complexes were not detected in the initial HOKs in the presence of Dynasore. As the result, the complexes were also not observed in the CellTracker labeled HOKs. These results demonstrate a potentially important role of *P. gingivalis* vesicles in facilitating mucosal transmission of HIV.

We next tested if the *P. gingivalis* vesicle-mediated internalization of HIV_NL4.3_ in HOKs can be transferred to permissive cells expressing HIV receptors. A TZA assay, a reporter cell-based assay for quantifying HIV-1 infection^19^, was performed to determine the role of *P. gingivalis* vesicles in HIV translocation. We first seeded HOKs in a 96-well plate, then added HIV_NL4.3_ alone or HIV-*P. gingivalis* 33277 vesicle complexes. After 1 h incubation, extracellular HIV bacterial vesicles were removed, and the HOKs were co-cultured with TZM-bl cells for 48 h. β-gal activity was measured using a TZA assay. Some enhancement of β-gal activity was found in the wells with HOKs exposed to HIV-1 alone, which may be due to incomplete removal of extracellular HIV-1. Interestingly, a 4.5-fold higher of β-gal activity was observed in the wells with HOKs exposed to HIV-vesicle complexes compared to that in the wells with cells exposed to HIV alone, after β-gal levels were normalized with the background found in the wells with untreated cells (Table 1). HIV infectivity was significantly blocked when HOKs were treated with dynasore (30 µM), which was consistent with our data on HIV-vesicle spreading among HOKs. These data indicate that a *P. gingivalis* vesicle-induced and endocytosis-dependent pathway is involved in this HIV-1 transmission between epithelial cells and HIV-1 permissive cells.

**Table 1:** *P. gingivalis* 33277 vesicle-dependent HIV_NL4.3_ translocation from HOKs to TZM-bl cells.

HOKs treated by	β-Gal (RLU) × 10^3 a^	Fold change compared to untreated HOKs	*P*-value
Untreated HOKs	4.40 ± 0.17		
HOKs + Vesicles	4.83 ± 0.37	0.002	0.17
HOKs + HIV	8.24 ± 0.32	1.87	0.013*^b^
HOKs + HIV/Vesicles	22.8 ± 3.73	5.18	0.007*
HOKs + dynasore + HIV	5.16 ± 1.05	1.17	0.246
HOKs + dynasore + HIV/Vesicles	5.96 ± 0.01	1.35	0.001*

^a^HOKs were first exposed to HIV_NL4.3_ or HIV-vesicle complexes, then mixed and cultured with TZM-bl cells for 48 h. A TZA assay was used to quantify inducible, replication-competent HIV-1 in HOKs.

^b^Asterisks indicate statistical significances of β-gal a ctivity in the wells with TZM-bl cells co-cultured with treated HOKs and untreated HOKs (*P* < 0.05; *t* test).

**Figure 7:**
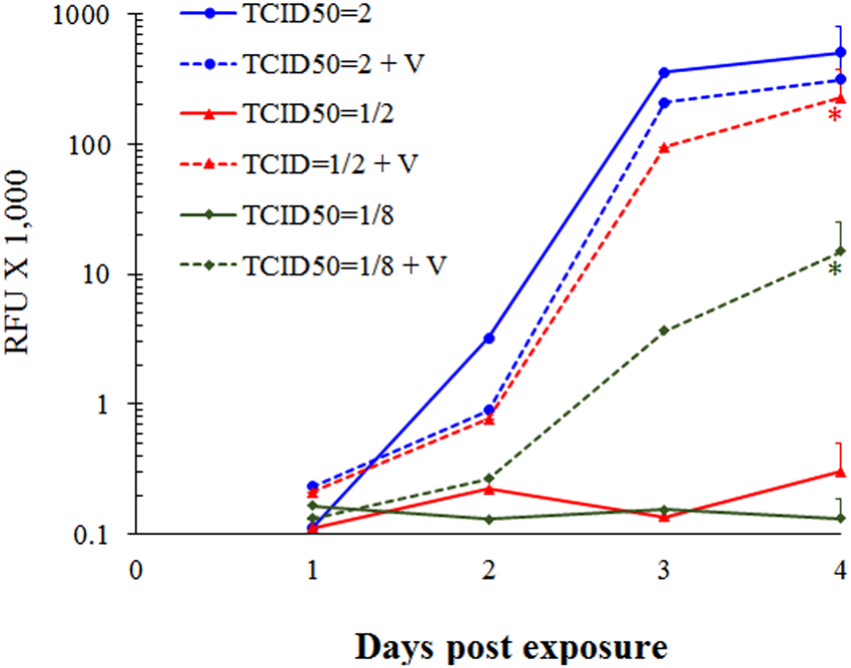
*P. gingivalis* vesicles enhanced HIV-1 infection of MT4 cells. MT4 cells (1 × 10^5^ cells) were seeded in 96-well plates and infected with NL4-3-SecNluc at TCID50 = 2, 1/2, and 1/8 per well in the presence (dashed lines) or absence (solid lines) of *P. gingivalis* vesicles (V) at 1/2 µg/ml. After infection, the culture supernatants were collected every day for four days. The luciferase activity (expressed as RFU × 1,000), used as a surrogate for virus replication, was measured with a PerkinElmer Victor II luminometer. Asterisks indicate significant differences between the infectivity levels detected in MT4 cells infected with HIV alone and HIV-vesicle complexes, respectively (*P* < 0.05, *t* test).

To test if *P. gingivalis* vesicles can also enhance HIV-1infectivity of permissive cells, HIVNL4.3-SecNluc-Sec infection was performed in the presence or absence of vesicles derived from *P. gingivalis* 33277. HIVNL4.3-Nluc-Sec NL4.3-SecNluc is a reporter virus with the secretory nanoluc gene inserted into the nef gene of the HIVNL4.3 genome 22. MT4 cells (human CD4 + T lymphoblastoma cells) were infected with a series of low infecting doses of NL4-3-SecNluc ranging from 1/8 to 2 TCID50. As expected, the virus infected MT4 cells at 2 TCID50 with or without the vesicles (Fig. 7). In the absence of vesicles, we did not observe NL4-3-SecNluc replication in MT4 cells infected with lower multiplicities of infection (1/2 or 1/8 TCID50/well of the virus) under the experimental conditions (Fig. 7). In contrast, viruses at the same low infecting doses successfully infected MT4 cells in the presence of the vesicles. These results indicate that *P. gingivalis* vesicles enhanced the infectivity of NL4-3-SecNluc by at least 10-fold. This enhancement was likely due to the ability of *P. gingivalis* vesicles to carry the virions into MT4 cells.

## Discussion

The association of HIV infection and mucosal microbiome, especially at the intestinal and genital surfaces, has been intensely investigated in recent years^20^. Studies have shown that decreased levels of *Lactobacillus*, associated with vaginosis, correlates with a higher susceptibility to HIV infection as well as with a decreased effectiveness of HIV treatment in women in the KwaZulu-Natal province of South Africa^21,22^. A mounting body of evidence suggests that the relationship between the microbiome and HIV infection may be a mutually-beneficial one for the pathogens^23^. Moreover, the microbiome, including microbes and their products, can induce local and systemic inflammation and immune activation. The persistent immune activation appears to be associated with enhanced morbidity and mortality of HIV/AIDS patients^20^. On the other hand, HIV-1 infection may lead to alterations in the composition of the gut microbiota, such as a decrease in *Lactobacillus* levels, which may cause structural damage to the gastrointestinal tract^24^. Previously, we reported a novel mechanism of HIV entry into non-permissive cells, in which *P. gingivalis*, an invasive oral bacterium, interacts specifically with HIV-1 and promotes a CD4-independent HIV entry into epithelial cells. Here, we further demonstrated that OMVs derived from *P. gingivalis* also play an important role in HIV mucosal infection and transmission. These data may explain how HIV-1 breaches the epithelial barrier and reaches HIV permissive cells, such as T-cells and dendritic cells. Based on our findings, we propose that invasive microbes and their outer membrane vesicles capable of specifically interacting with HIV-1 may serve as a “vehicle/carrier” that translocates HIV across the mucosal epithelia. Findings from previous studies suggest that intracellular *P. gingivalis* exits the gingival epithelial cells via a recycling pathway, and that knockdown of Rab11, RalA and exocyst complex subunits significantly prevents *P. gingivalis* from exiting the infected cells^25,26^. Therefore, it is possible that the translocation of HIV-vesicle complexes also exploits a similar mechanism.

Based on our previous observations that *P. gingivalis* is able to enter, exit and reenter HOKs^18^, and that *P. gingivalis* vesicles can translocate through monolayered HOKs^13^, we hypothesized that *P. gingivalis* vesicles are capable of carrying HIV-1 from cells to cells and spreading among HOKs. The current study provides solid evidence to support *P. gingivalis* vesicles-mediated HIV-1 transcytosis. Several studies have reported cell-free HIV transcytosis through intestinal, vaginal, genital, and oral epithelial cells, although the mechanism has not been elucidated^27–29^. A recent report showed that HIV-1 entered oral and genital epithelial cells and became sequestered in the endosomes for as long as 9 days^30^. The authors of this study also revealed that the release of the sequestered HIV-1 from endosomes was induced by an increase in the intracellular calcium level and the disruption of cortical actin in the epithelial cells. Here, we discovered a rapid HIV entry into and translocation among HOKs, which is mediated the bacterial outer membrane vesicles. Intracellular HIV-vesicle complexes were observed within 20 minutes after initial exposing the HOKs to the HIV-vesicle complexes. Translocation of HIV-1 among HOKs was found within 24 h of exposure in the presence of *P. ginigvalis* vesicles, and spreading HIV-1 from HOKs to CD4^+^ cells within 48 h. These data present a novel mechanism of efficient HIV-1 entry into and spread among non-permissive cells. Moreover, we also showed that some of intracellular HIV-1 could be reverse-transcribed and integrated into the genome of HOKs within 1 h. It is likely that the reverse-transcription occurred when HIV-1 had freed from *P. gingivalis* vesicles, although the mechanism(s) of HIV-1 dissociating from *P. gingivalis* vesicles and releasing to cytoplasm of HOKs is currently not known. A number of intracellular pathogens such as *Listeria*, *Rickettsia*, *Shigella*, and *Burkholderia* are internalized in a vacuole^31,32^. The bacteria rapidly escape from the vacuole after invasion, and free bacteria in the cytosol can be detected within 30 minutes after invasion. The mechanism of *Listeria monocytogenes* escape involves in the bacterial enzyme listeriolysin O that inserts into the vacuolar membrane by binding cholesterol, which leads to pore formation and membrane interruption^33,34^. It is found that vacuole acidification is a trigger for bacterial escape from the vacuole, as listeriolysin O is optimally active only in an acidic environment (pH 5.5)^35^. Conditions requiring for escape of HIV-1 and/or HIV/vesicle complexes from endosomes are now under investigation in our laboratories. In general, HIV-1 entry into the permissive cells occurs 1–3 h after infection. Reverse transcription initiates over the 6 to 48 hours, and viral genome integration takes place a few hours after reverse transcription^36,37^. Compared to conventional routes of HIV-1 infection, the vesicle-mediated viral entry, reverse transcription, and integration appear to occur at a much faster pace. We previously reported that *P. gingivalis* vesicles enter host cells in less than 15 minutes^13^, which may contribute to more rapid reverse-transcription and integration observed in the HOKs. However, the mechanism(s) responsible for the fast kinetic of viral life cycle mediated by the vesicle remains to be determined.

In addition to their ability to promote receptor-independent HIV-1 entry into non-permissive cells, *P. gingivalis* vesicles also enhanced HIV infection of T-cells at low doses that otherwise could not initiate a productive infection in the absence of the vesicles. Presumably, by binding to *P. gingivalis* vesicles, HIV-1 particles, even at non-infectious doses, can now enter permissive cells using the vesicles as a vehicle. It was previously estimated that only 0.01–1% of HIV-1 particles in a culture sample are infectious^38–45^. In other words, the majority of virions cannot infect HIV-1 susceptible cells due to defects in their viral proteins or genes. Some of the non-infectious viral particles may have aged or are abnormally assembled HIV-1 Env trimers that are compromised in viral entry. A specific interaction between *P. gingivalis* vesicles and HIV-1 may enable such non-infectious virions to enter CD4^+^ cells through an endocytic pathway^46^. Once inside the cells, HIV-1 with non-lethal mutations can start a new life cycle and produce infectious progenies. Therefore, our findings suggest that *P. gingivalis* vesicles not only promote HIV translocation from mucosal surfaces to the subcutaneous tissues, but also enhance HIV infection of permissive cells.

The mechanism by which OMVs facilitate HIV entry into human oral epithelial cells may also be relevant to HIV transmission across the genital, rectal, and vaginal mucosal epithelia. This is because a wealth of microbial flora is also present in these areas, including Gram-negative invasive bacteria such as the *Prevotella* and *Porphyromonas* species. Hence, manipulating the mucosal microbiome and disrupting bacterial invasion may have an impact on preventing mucosal transmission of HIV-1.

## Methods

### Bacterial strains and vesicle preparation and quantification

*P. gingivalis* 33277 was grown from frozen stocks in trypticase soy broth (TSB) or on TSB blood agar plates supplemented with yeast extract (1 mg/mL), hemin (5 μg/mL) and menadione (1 μg/mL), and incubated at 37 °C in an anaerobic chamber (85% N2, 10% H2, 5% CO2). *P. gingivalis* vesicles were prepared as previously described^46^. Briefly, *P. gingivalis* was grown to the late exponential phase, and growth media were collected by centrifugation at 10,000 × *g* for 15 minutes at 4 °C and filtered through 0.22-μm-pore-size filters (Cell Treat, MA) to remove residual bacteria. Vesicles were collected by ultracentrifugation at 126,000 × *g* for 2 h at 4 °C and resuspended in phosphate-buffered saline (PBS) containing 10% glycerol. OMVs were quantitated using both protein and lipid assays. Proteins and lipids were extracted from vesicles in PBS using a BugBuster® Protein Extraction Reagent (Novagen, MA).

### Cell culture and HIV production

Human oral keratinocytes (HOKs) were obtained from ScienCell Research Laboratories (Carlsbad, CA) and cultured in the oral keratinocyte medium recommended by ScienCell. TZM-bl cells were obtained from the NIH AIDS Reagent Program and cultured in Dulbecco’s modified Eagle’s medium (DMEM) (Invitrogen, Grand Island, NY) supplemented with 10% heat-inactivated fetal bovine serum, penicillin (100 U/mL), and streptomycin (100 µg/mL) at 37 °C in 5% CO2. X4-tropic HIV-1_NL4–3_ and R5-tropic HIV-1_YU2_ were produced by transfecting 293 T cells with pNL4-3 or pYU2 (AIDS Research and Reference Reagent Program, NIAID) using the FuGENE 6 transfection reagent (Promega, Madison, WI). The viral supernatants were collected at 48 h post-transfection, and stored in aliquots at −80 °C. The viral stocks were quantified by measuring p24 antigens using an ELISA kit (PerkinElmer Life Sciences, MA)^10^.

### Examination of HIV-1 and *P. gingivalis* vesicle interactions

*P. gingivalis* vesicles were coupled with Dynabeads (Life Technologies, CA) according to the manufacturer’s instructions. Briefly, 20 µl of Dynabeads (8 × 10^6^) were washed with 1 ml of Buffer 1 (0.1 M sodium phosphate buffer, pH 7.4–8.0) and resuspended in 500 µl of Buffer 1. The Dynabeads were then coupled with 500 ng of the vesicles for 30 minutes. Bovine serum albumin (BSA, 1% w/v) was added to block the free epoxy groups on the beads. The beads were then washed three times with 1 ml of Buffer-2 (PBS supplemented with 0.1% BSA and 2 mM EDTA, pH 7.4) and resuspended in 500 µl of Buffer-2. HIV-1 (NL 4.3 or YU2, 10 ng of p24) was then incubated with bead for 1 h. The beads were then washed three times with PBST (PBS supplemented with 0.02% Tween-20) to remove unbound viruses. The beads were then fixed with 3.8% formaldehyde, permeabilized with 0.1% Triton-X 100, and blocked with 3% BSA in PBS for 1 h. The beads were then stained with a mouse anti-p24 monoclonal antibody (NIH AIDS Reagent Program) and pan-specific rabbit anti *P. gingivalis*, followed by Alexa Fluor 488-conjugated chicken anti-mouse IgG and Alexa Fluor 546-conjugated donkey anti-rabbit IgG (Life Technologies). The beads were mounted on a glass slide with ProLong® Diamond Antifade Mountant (Thermo Fisher Scientific, MA). Confocal images were acquired using a Nikon A1R confocal microscope.

### *P. gingivalis* vesicles mediated HIV-1 entry

*P. gingivalis* vesicles were incubated with HIV_NL4.3_ for 15 minutes to facilitate the formation of HIV-vesicles complexes. HOKs (4 × 10^5^ cells) were grown overnight on glass-bottom microwell dishes (MatTek, MA) and were exposed to *P. gingivalis* 33277 vesicles, HIV_NL4.3_ or HIV (10 ng of p24)/vesicle (50 ng) complexes for 0.5 or 1 h. The infected cells were fixed with 3.8% formaldehyde, permeabilized with 0.1% Triton X-100, and blocked with 5% bovine serum albumin. *P. gingivalis* vesicles were stained with rabbit polyclonal FimA antisera followed by goat anti-rabbit Alexa Flour 546-conjugated antibodies (Life Technologies). Confocal images were acquired using a Nikon A1R confocal microscope.

Live-cell imaging of HIV entry was performed using an A1R confocal microscope equipped with a heated cell chamber supplemented with 5% CO2. Briefly, *P. gingivalis* 33277 vesicles were labeled using the Alexa Fluor 594 Microscale Protein Labeling Kit (Thermo Fisher Scientific). The labeled vesicles were collected and washed twice with PBS by centrifugation at 28,000 rpm for 2 h. The labeled vesicles were then incubated with green fluorescent protein (GFP)-tagged HIV (Vpr-GFP-tagged NL4.3, 10 ng of p24) for 15 minutes. HOKs were first cultured on 35 mm glass bottom dishes for 16 h and then exposed to HIV_NL4.3_ alone or HIV/vesicle complexes and mounted on the 37 °C microscope stage. Images were collected using a 40×/1.3-numerical aperture (NA) oil platform objective at 10-sec intervals using Nikon Advanced Research Imaging Software (Nikon Instruments).

### Quantitative real-time PCR (qPCR)

DNA was extracted using an Easy DNA^TM^ Kit (Life Technologies). Real-time qRT-PCR was conducted using a QuantiTect SYBR Green RT-PCR kit (Qiagen, Germany) on an iCycler MyiQ^TM^ Real-Time PCR detection system (Bio-Rad Laboratories, Inc, CA) according to manufacturer’s instructions. Viral DNA was quantified with HIV *gag-* and *ltr-* specific primers (Table S1)^11^. Human glyceraldehyde 3-phosphate dehydrogenase gene (*gapdh*) was used as a normalizing gene. The melting curve profile was analyzed to verify a single peak for each sample. The levels of *gag* and *ltr* genes for the test sample were determined relative to the untreated calibrator sample by using the comparative cycle threshold (*ΔΔC*_*T*_) method.

### Alu-LTR real-time nested PCR assay

Integration of viral DNA into the genomic DNA of HOKs was determined using an Alu-long terminal repeat (LTR)-based real-time nested-PCR assay^47^. Two human genomic Alu forward primers (Alu1 and Alu2) and an HIV-1 LTR reverse primer extended with a lambda phage-specific heel sequence at the 5′ end of the oligonucleotide (M667–L) were used to enrich host genomic DNA with downstream proviral DNA using PCR. The integrated HIV-1 provirus was then quantified using an iQ Supermix kit (Bio-Rad) with a reaction solution containing a lambda-specific primer (Lambda T) and an *ltr* primer (AA55M) (Table S1), and the PCR products from the first-round of PCR. Amplification of human *gapdh* gene was used for normalizing levels of HIV-1 provirus.

### Examination of HIV spreading among HOKs

HOKs were exposed to either HIV_NL4.3_ alone or HIV/vesicle complexes for 30 minutes. The exposed HOKs were collected by trypsinization, washed two times with PBS, and mixed with a new set of uninfected HOKs labeled with CellTracker Blue CMAC (Invitrogen). The mixed HOKs were seeded in glass-bottom microwell dishes and cultured for 24 h. These cells were then immunostained with anti-*P. gingivalis* polyclonal antibodies to detect *P. gingivalis* vesicles, and anti-viral p24 to detect HIV. Confocal microscopy images were obtained using a Nikon A1R confocal microscope.

### TZA assay

TZA assay was performed as previously described with some modifications^19^. HOKs (2 × 10^4^ cells) were seeded in four replicates in a 96-well plate and first cultured in oral keratinocyte medium to 70% confluence. HOKs were then exposed to *P. gingivalis* vesicles, HIV_NL4.3_ alone, or HIV-vesicle complexes for 30 minutes. After five washes with PBS, TZM-bl cells (1.5 × 10^4^ cells) were added to each well, and both cells types were co-cultured in DMEM containing 10% FBS for 48 h. The wells were washed, and Beta-Glo reagent (Promega, Madison, WI) was added and incubated with the cells for 1 h. Chemiluminescence was measured using a FLUOstar Microplate Reader (BMG Labtech, Cary, NC).

### Multi-cycle viral replication in MT4 cell assay

HIV-1 NL4-3 Nanoluc-sec at various low infecting doses (ranging from 1/8 to 2 TCID_50_/well) was used to infect MT4 cells (1 × 10^5^ cells/mL) in the presence or absence of HIV_NL4.3_ or HIV-vesicle complexes in 96-well plates. Supernatant samples were harvested on day 3 post-infection and assayed for luciferase activity using the Nano-Glo® Luciferase Assay System (Promega). A relative Luminescence level 10-time higher than background (cell only) was scored as a productive viral infection.

### Statistical analyses

A student’s *t*-test was used to determine the statistical significance of the differences in HOK response to exposure to HIV-1 alone or HIV-*P. gingivalis* vesicle complexes. *P* < 0.05 was considered significant. Values shown are ± SD unless stated otherwise.

## Electronic supplementary material

Live image of P. gingivalis mediated HIV entering HOKs.

Live image of P. gingivalis mediated HIV entering HOKs in the presence of dynasore.

S1 Table. Oligonucleotide primers used in this study
