# Supplementary material for: Role of Porphyromonas gingivalis outer membrane vesicles in oral mucosal transmission of HIV
Source: Sci Rep. 2018 Jun 11;8:8812. doi: 10.1038/s41598-018-27284-6 (PMC5995904; doi:10.1038/s41598-018-27284-6)
Supplement: Supplementary file 3 — S1 Table. Oligonucleotide primers used in this study [file 41598_2018_27284_MOESM3_ESM.doc]

**Role of *Porphyromonas gingivalis* outer membrane vesicles in oral mucosal transmission of HIV**

Xin-Hong Dong ^1^, Meng-Hsuan Ho^2^, Bindong Liu^1^, James Hildreth^1^, Chandravanu Dash^3^, J Shawn Goodwin^3^, Muthukumar Balasubramaniam^3^, Chin-Ho Chen^4*^, and Hua Xie^2*^

**S1 Table. Oligonucleotide primers used in this study**

| Gene | Primer name | Primer sequences (5’-3’) |
| --- | --- | --- |
| *gag* | Gag-162F  Gag-162R | CCCATAGTGCagaacatcca  gggctgaaagccttctcttc |
| *ltr* | ltr s4  ltr S3 | AAGCCTCAATAAAGCTTGCCTTGA  GTTCGGGCGCCACTGCTAG |
| *Alu1* | Alu 1 | TCCCAGCTACTGGGGAGGCTGAGG |
| *Alu2* | Alu 2 | GCCTCCCAAAGTGCTGGGATTACAG |
| *Lambda T* | LambdaT | ATGCCACGTAAGCGAAACT |
| *M667* | M667 | GGCTAACTAGGGAACCCACTG |
| *AA55M* | AA55M | GCTAGAGATTTTCCACACTGACTAA |
| *gapdh* | GAPDH-126F  GAPDH-126R | gttgctgtagccaaatcgttgt  ggtggtctcctctgacttcaaca |
